# Spontaneous omental infarction as a rare differential for right iliac fossa pain: A case report and review of the literature

**DOI:** 10.1002/ccr3.9151

**Published:** 2024-07-03

**Authors:** Su Jin Lee, Khang Duy Ricky Le, Peter Mark

**Affiliations:** ^1^ Department of General Surgical Specialties The Royal Melbourne Hospital Melbourne Victoria Australia; ^2^ Department of Radiology The Royal Melbourne Hospital Melbourne Victoria Australia; ^3^ Department of Surgical Oncology Peter MacCallum Cancer Centre Melbourne Victoria Australia; ^4^ Geelong Clinical School Deakin University Geelong Victoria Australia; ^5^ Department of Medical Education, Melbourne Medical School The University of Melbourne Melbourne Victoria Australia

**Keywords:** abdominal pain, computer tomography, infarcted omentum, spontaneous infarcted omentum

## Abstract

Omental infarction is a rare cause of acute abdominal pain, often benign and self‐limiting. The significance of infarction lies in the fact that it can mimic other abdominal pathologies including appendicitis, cholecystitis, pancreatitis, or reflux disease. Diagnostic laparoscopy provides the definitive diagnosis of omental infarction, but it is invasive and limited due to resources. Computed tomography of the abdomen and pelvis has been considered the gold standard to diagnosing omental infarction when a non‐invasive diagnostic approach is required. Additionally, ultrasound can also be used alternatively for children. Currently, there is no consensus in the diagnosis and management of patients with imaging‐proven omental infarction. Spontaneous infarcted omentum must be considered by surgeons and radiologists as a rare cause of acute abdominal pain as patients can experience good outcomes with either conservative or operative approach. However, conservative management must only be considered in stable patients where alternative pathology is unlikely.

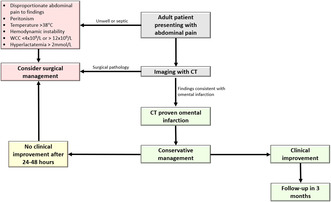

## 1 BACKGROUND

Omental infarction is a rare diagnosis that may occur in patients with acute or subacute abdominal pain.^1^, ^2^, ^3^, ^4^ Despite its low incidence, omental infarction may mimic common surgical presentations including acute appendicitis, acute cholecystitis, and diverticulitis. Furthermore, clinical examination and biochemical investigation of patients with proven omental infarction lack sensitivity and specificity for the condition, with diagnosis requiring computer tomography (CT) imaging.^5^ Currently, there remains no evidence‐based framework in the approach and management of omental infarction. In particular, current approaches to omental infarction remain highly heterogeneous, with good outcomes following a conservative non‐operative approach identified in the literature as well as with operative intervention.^6^ Despite these similar outcomes, more often, patients are diagnosed and managed following surgical interventions such as via laparoscopy or laparotomy to address potential acute surgical conditions that omental infarction can mimic, such as appendicitis and cholecystitis. These procedures within themselves carry risk, and in the context of omental infarction may not necessary. In this case report, we highlight the presentation of a 23‐year‐old female with right iliac fossa pain suspicious for appendicitis with a laparoscopy demonstrating omental infarction which was resected. We then evaluate the current literature to explore best‐practice approaches to the diagnosis and management of this condition.

## 2 CASE PRESENTATION

The patient provided written consent for the de‐identification and use of their medical information and data for the generation and publication of this case report.

### 2.1 Case history and examination

A 23‐year‐old Chinese international student presented to the emergency department with 3 days of gradual onset right iliac fossa abdominal pain. Her pain was constant in nature and exacerbated by movement. Her past medical history included left inguinal hernia repair as a child. She was not on any regular medications. She was a non‐smoker and a social drinker. She was up to date with sexually transmitted infections screening. She was in the middle of her menstruation cycle and reported heavy menstrual bleeding during these periods without any per vaginal discharge. On examination, her abdomen was soft and tender in the right lower quadrant with no signs of peritonism.

### 2.2 Methods (differential diagnosis, investigations, and treatment)

**FIGURE 1:**
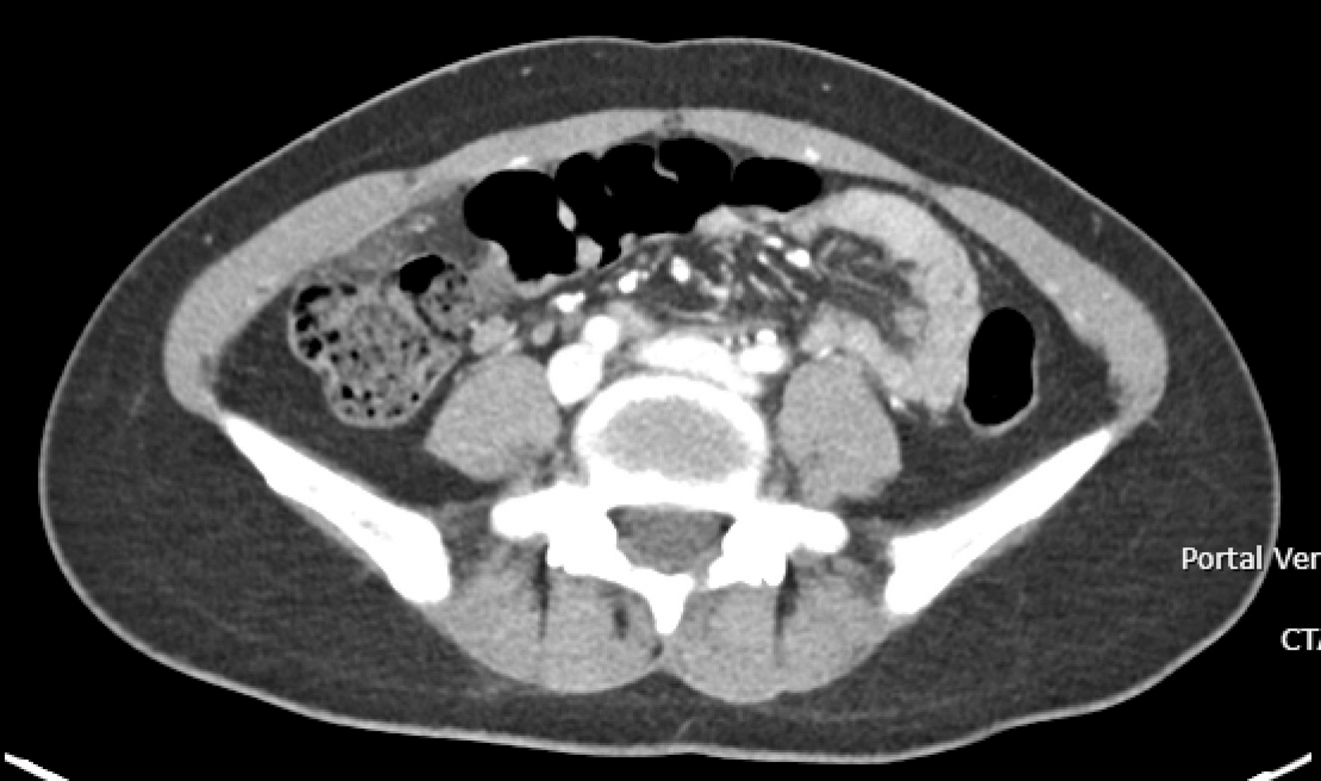
Axial View of CT abdomen/pelvis demonstrated inflammatory fat stranding suggestive of omental infarction.

**FIGURE 2:**
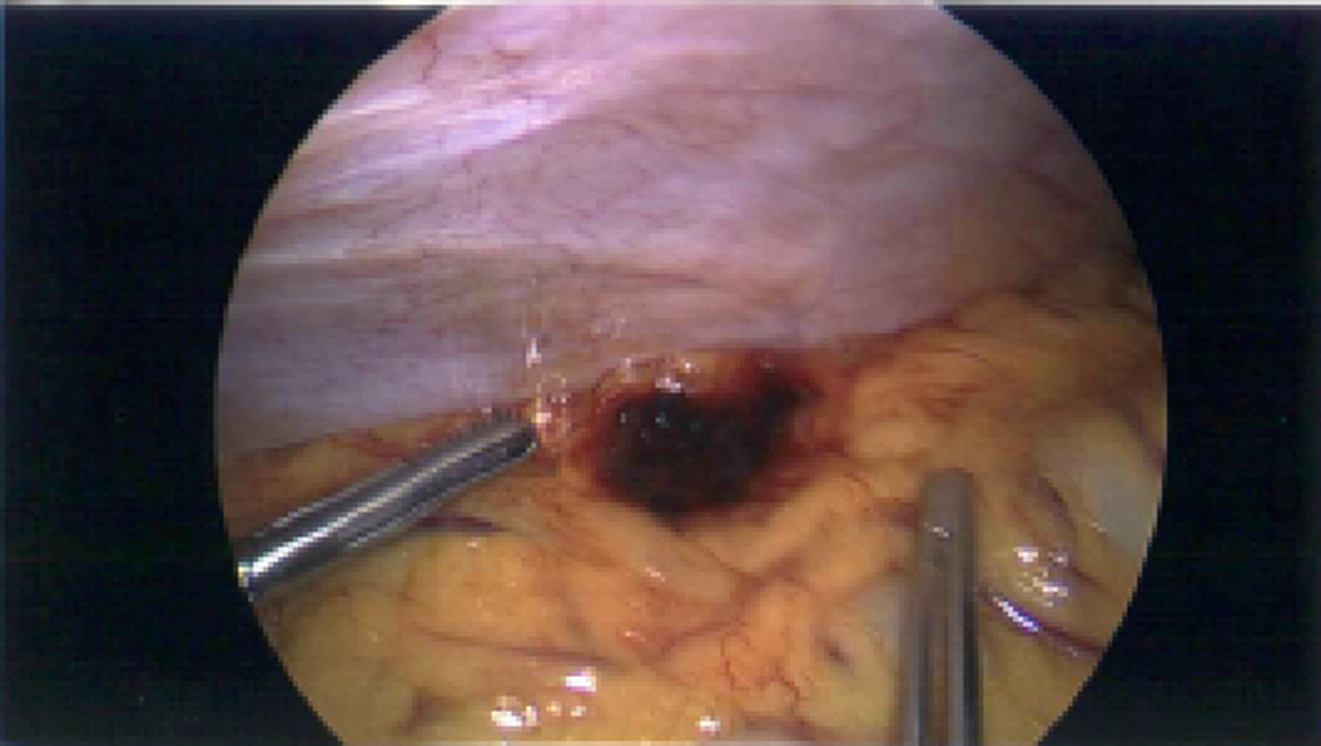
Intraoperative finding of omental infarction.

Her laboratory investigations were significant for a hemoglobin (Hb) of 78 g/L, white cell count (WCC) of 7.7 × 10^9^/L and C‐reactive protein (CRP) of 0.8 mg/L. Her liver and kidney function tests were unremarkable. CT of the abdomen and pelvis demonstrated a slightly thickened appendix which measured up to 8 mm without definitive inflammatory change, and subtle inflammatory fat stranding anterior to the caecum which was consistent with the diagnosis of omental infarction (Figure 1). She was admitted for observation and pain management with regular paracetamol, ibuprofen and oxycodone‐naloxone 5–2.5 mg twice a day, as well as oxycodone 5 mg as required for breakthrough. Her subsequent blood tests remained unremarkable with Hb of 78 g/L, WCC of 5.0 × 10^9^/L and CRP of 0.7 mg/L. Despite her biochemical and vital sign stability, the patient continued to experience pain. On day 2 of admission, the decision was made to proceed with diagnostic laparoscopy. Intra‐operative findings confirmed infarcted piece of omentum, which was adhered to the anterior abdominal wall (Figure 2). Additional findings included trace amounts of blood in the pelvis and otherwise normal appendix, ovaries, uterus, small bowel, and large bowel. Excision of infarcted omentum was performed with diathermy and endoloop for hemostasis.

### 2.3 Conclusion and results (outcome and follow‐up)

Following surgery, the patient's pain resolved and she was discharged on the subsequent day. The pathology of the excised tissue confirmed omentum with acute ischemic change. She was followed up 2 weeks post discharge and made a good recovery.

## 3 DISCUSSION AND ALGORITHM FOR MANAGEMENT

Omental infarction is a rare cause of acute abdominal pain and most prevalent in patients aged between 30 and 50 years.^6^ Furthermore, there is a clear predominance in male (2:1 ratio compared to females) and obese populations.^6^ In the literature, approximately 400 cases have been described, however there remains a significant paucity of evidence to robustly evaluate the true prevalence and incidence of this condition. Omental infarction is heterogeneous in its presentation, ranging from localized acute or subacute abdominal pain, often with localized tenderness at the site of infarction on examination.^7^ Cases studies have identified that the timeframe of pain onset is also highly variable; ranging from a day prior to hospital presentation to months prior.^8^ Biochemically, non‐specific inflammatory responses are noted including mild leucocytosis, elevated erythrocyte sedimentation rate (ESR), and elevated CRP.^7^, ^9^ Omental infarction can therefore mimic the classical presentation of the acute abdomen and is often misconstrued for common pathologies including appendicitis, cholecystitis, pancreatitis, reflux disease as well as gynecological presentations such as ovarian cyst rupture due to its non‐specific presentation. Furthermore, given the rarity of the condition, omental infarction is often not at the forefront of the surgical treating team when considering differential diagnoses for the acute abdomen, with poorly standardized approaches to diagnosis and management.

The pathophysiology of omental infarction remains unclear. The omentum is a fold of visceral peritoneum, extending from the stomach to adjacent organs and anatomically divided into the greater and lesser omentum.^10^ 90% of cases occur in the right side of the omentum due to greater length, mobility, and limited blood supply.^11^ It can be idiopathic or secondary to torsion, hernia, adhesions, trauma, vascular disease, or hypercoagulability.^12^ Patients with omental infarction have been reported in the literature to be often diagnosed intra‐operatively following abdominal surgery (diagnostic laparoscopy or laparotomy) performed to diagnose and manage more common surgical pathologies such as cholecystitis and appendicitis.^13^ Therefore, there remains significant limitations in the diagnosis of this condition using clinical examination and biochemistry alone. However, with advancement in radiological technologies, CT scan of the abdomen and pelvis is becoming a more reliable investigation to diagnose omental infarction. The well‐established radiological characteristic of omental infarction is fat stranding adjacent to the bowel that is disproportional to the thickening of the bowel wall. CT also plays another important pivotal role in the diagnostic approach for omental infarction, namely through the exclusion of other important causes of the acute abdomen such as pancreatitis, cholelithiasis and cholecystitis, appendicitis and colitis. However, there remains heterogeneity in the way that infarcted omentum displays on CT, with a range from well‐defined fat density lesions with hyperdense rim enhancement to ill‐defined hyperdensities.^14^ Given this, the radiologist must retain a high index of suspicion for omental infarction, particularly when the clinical history does not correlate with abdominal findings on imaging, such as in the context of normal appendix and normal bowel. Despite the adult predominance, it is important to recognize that omental infarction also occurs in children with prevalence of 0.1%–0.5% and therefore, such diagnoses should remain on the radar for radiologists and surgeons alike.^15^ In children, ultrasound is an alternative imaging modality to delineate omental infarction with an appearance of noncompressible and hyperechogenic mass in the omentum.^4^

The management of omental infarction remains controversial. The general experience is that most of cases should be treated conservatively through oral analgesia, anti‐inflammatories, and antibiotics in select cases. Conservative approaches are generally favorable as it avoids surgical and anesthetic risks. This is a particularly favored approach in children to avoid the risks of upfront surgery.^16^ Furthermore, the literature suggests that in this population, the natural history of this condition is benign and self‐limiting, often resolving within 10–15 days.^17^, ^18^ For adults, it is suggested that conservative management be trialed for shorter periods, such as between 24 and 48 h, with an argument that remains for surgical intervention outside of this to reduce the incidence of omental necrosis and lower the risk of developing intra‐abdominal collections or adhesions.^13^ Furthermore, conservative strategies often have a protracted length of time towards resolution that can range from days to weeks, with surgical management allowing faster resolution of symptoms and shorter hospitalization.^6^ Despite this, there is noticeable heterogeneity in approach to the management of those with omental infarction. In the literature, 27.2% of patients are described to fail conservative management and proceed to laparoscopic or open surgery. Therefore, for treating teams, the dilemma lies in stratifying patients into whether they would most benefit from a conservative or operative approach. Patients who were febrile at presentation and with WCC greater than or equal to 12 × 10^9^/L have been described to likely fail conservative management.^6^ This highlights the fact that patients with signs and symptoms suggestive of infection, sepsis, or instability warrant consideration of early surgical intervention for source control and definitive diagnosis of causes of the acute abdomen, in addition to the aforementioned improved outcomes in symptoms and length of stay. However, as a more invasive approach, the risks of surgical intervention must also be considered. These include the peri‐operative risks of anesthesia, development of intra‐operative complications such as bleeding, infection, damage to surrounding structures and surgical mortality. Given the lower acuity and often self‐limiting nature of omental infarction in children, these surgical risks may urge clinicians to have a higher threshold for surgery and to therefore adopt conservative approaches. For the adult patient, shared‐decision making following judicious clinical assessment is warranted to balance the risks of surgery versus conservative management.

**FIGURE 3:**
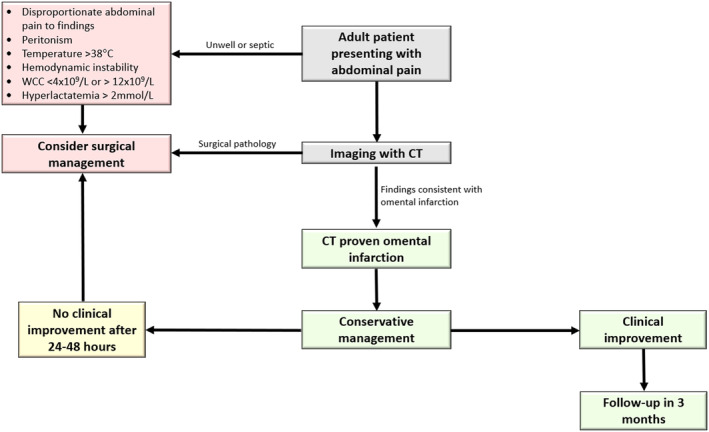
Proposed algorithm for management of CT proven omental infarction in the adult population.

Overall, there are no current guidelines established in management of omental infarction. The choice of management has been largely on an individual basis, with consideration of the clinical picture, investigations, age and comorbidities. Based on the current literature, our case report and literature review propose a safe step‐up treatment approach for patients with CT‐diagnosed omental infarction (Figure 3). For patients who are over 18 years of age and present with disproportional abdominal pain and signs of peritonitis, haemodynamic instability, temperature greater than or equal to 38 degrees Celsius, WCC less than 4 × 10^9^/L and greater than 12 × 10^9^/L, or hyperlactatemia greater than 2 mmol/L, surgical management should be strongly considered given the higher chance of alternate pathology. For patients who does not meet the criteria, conservative management should be considered in first 24–48 h of presentation with oral analgesia including nonsteroidal anti‐inflammatory medications and fluid management. In such patients, if there is no clinical improvement in first 24–48 h, surgical management should be considered. This is suggested to evaluate for alternate pathology to explain the failure to progress and thrive. Furthermore, it is important to assess for the complications of omental infarction in these patients including abscess formation, adhesions or recurrent infarction.^6^ Similarly, those managed conservatively should be followed up 3 months post discharge to assess for the same.^7^ Overall, our case report highlights that that omental infarction should be considered, despite its relative rarity, in the differential diagnosis of acute abdominal pain. Furthermore, omental infarction should particularly be considered in more nuanced cases of abdominal pain where the clinical presentation does not entirely fit with what is expected of common conditions including appendicitis and cholecystitis. From our case and evidence from the literature, our proposed algorithm highlights a safe approach to managing patients with infarcted omentum, balancing the need for surgery with more conservative management approaches. There however remains relatively poorly characterized evidence given the rarity of omental infarction, with a need for more prospective trials with larger sample sizes to derive more robust evidence regarding outcomes following conservative or operative management of omental infarction to guide the development of standardized frameworks and guidelines.

## 4 CONCLUSION

Omental infarction presents a diagnostic challenge, with no established guidelines in management of omental infarction. Our case report and literature review highlights the need for radiologists and surgeons to be aware of this condition as an alternate pathology driving presentations of the acute abdomen. Our proposed treatment algorithm provides a structured framework for clinicians, emphasizing early consideration of surgical intervention in select cases and initial conservative management in others. Close monitoring and follow‐up are essential to detect complications in a safe and timely manner and to ensure optimal patient outcomes.

## AUTHOR CONTRIBUTIONS

**Su Jin Lee:** Conceptualization; data curation; formal analysis; investigation; methodology; project administration; resources; supervision; validation; visualization; writing – original draft; writing – review and editing. **Khang Duy Ricky Le:** Data curation; formal analysis; investigation; methodology; project administration; resources; supervision; validation; writing – original draft; writing – review and editing. **Peter Mark:** Conceptualization; formal analysis; supervision; writing – review and editing.

## FUNDING INFORMATION

There are no conflicts of interest or disclaimers. & editing. Peter Mark—Conceptualization, Formal analysis, Writing—review & editing.

## CONFLICT OF INTEREST STATEMENT

There was financial and grant support.

## ETHICS STATEMENT

The case report generation process was discussed with the Peter MacCallum Cancer Centre ethics and governance team. No formal ethics approval was required following the discussions. The patient provided written consent for the de‐identification and use of their medical information and data for the generation and publication of this case report.

## CONSENT

The patient provided informed consent that was written and signed for generation and publication of this manuscript using their de‐identified medical information.

## Data Availability

Data can be requested from corresponding author when required. All relevant data has been provided in the generation of this manuscript which is intended for open access publication.

## References

[1] Bin Mohamed Ebrahim ME , Kulasegaran S , Leibman S , Smith G . Greater omental infarction. ANZ J Surg. 2022;92(11):3080‐3081. 35174609 10.1111/ans.17545 PMC9790466

[2] Shin MK , Lee OJ , Ha CY , Min HJ , Kim TH . Malignant mesothelioma of the greater omentum mimicking omental infarction: a case report. World J Gastroenterol. 2009;15(38):4856‐4859. 19824125 10.3748/wjg.15.4856 PMC2761569

[3] Graham JA , Levinson SA . Omental apoplexy: idiopathic segmental infarction of the greater omentum. Am J Dig Dis. 1950;17(4):114‐117. 15410676 10.1007/BF03004918

[4] Tonerini M , Calcagni F , Lorenzi S , Scalise P , Grigolini A , Bemi P . Omental infarction and its mimics: imaging features of acute abdominal conditions presenting with fat stranding greater than the degree of bowel wall thickening. Emerg Radiol. 2015;22(4):431‐436. 25725796 10.1007/s10140-015-1302-0

[5] Gosain A , Blakely M , Boulden T , et al. Omental infarction: preoperative diagnosis and laparoscopic management in children. J Laparoendosc Adv Surg Tech A. 2010;20(9):777‐780. 20704515 10.1089/lap.2010.0204

[6] Medina‐Gallardo NA , Curbelo‐Pena Y , Stickar T , et al. Omental infarction: surgical or conservative treatment? a case reports and case series systematic review. Ann Med Surg. 2020;56:186‐193. 10.1016/j.amsu.2020.06.031 PMC7334794 32642061

[7] Breda Vriesman A , Lohle P , Coerkamp E , Puylaert J . Infarction of omentum and epiploic appendage: diagnosis, epidemiology and natural history. Eur Radiol. 1999;9:1886‐1892. 10602970 10.1007/s003300050942

[8] Park TU , Oh JH , Chang IT , et al. Omental infarction: case series and review of the literature. J Emerg Med. 2012;42(2):149‐154. 19097725 10.1016/j.jemermed.2008.07.023

[9] Shrestha B , Kumar R , Pandey S , Prasad AS , Dawra S . Spontaneous segmental infarction of the greater omentum. Journal of Medicine, Surgery, and Public Health. 2024;2:100073.

[10] Karak PK , Millmond SH , Neumann D , Yamase HT , Ramsby G . Omental infarction: report of three cases and review of the literature. Abdom Imaging. 1998;23(1):96‐98. 9437073 10.1007/s002619900294

[11] Soobrah R , Badran M , Smith SG . Conservative management of segmental infarction of the greater omentum: a case report and review of literature. Case Rep Med. 2010;2010:1‐4. 10.1155/2010/765389 PMC2945678 20886031

[12] Zhang AY , Griffin GM , Karrington BA , Tamura GS . Case report: a child with omental infarction. J Emerg Med. 2023;64(5):638‐640. 37032205 10.1016/j.jemermed.2023.02.008

[13] Buell KG , Burke‐Smith A , Patel V , Watfah J . Omental infarction: the great impersonator. Cureus. 2017;9:12. 10.7759/cureus.1940 PMC5811161 29468096

[14] Singh A , Gervais D , Lee P , et al. Omental infarct: CT imaging features. Abdom Imaging. 2006;31:549‐554. 16465576 10.1007/s00261-005-0251-6

[15] Siddiqui S , Ahmed A , Nadeem N . Omental infarction in a child. JAMC. 2016;28(3):623‐624. 28712252

[16] Kozlowski M , Piotrowska O , Gizewska‐Kacprzak K . Omental infarction in a child‐conservative management as an effective and safe strategy in diagnosis and treatment. Int J Environ Res Public Health. 2021;18(15):29. 10.3390/ijerph18158057 PMC8345747 34360347

[17] Arigliani M , Dolcemascolo V , Nocerino A , Pasqual E , Avellini C , Cogo P . A rare cause of acute abdomen: omental infarction. J Pediatr. 2016;176:216. 27289499 10.1016/j.jpeds.2016.05.034

[18] Rimon A , Daneman A , Gerstle JT , Ratnapalan S . Omental infarction in children. J Pediatr. 2009;155(3):427‐431. e1. 19540514 10.1016/j.jpeds.2009.03.039

